# Type I Interferon-Mediated Regulation of Antiviral Capabilities of Neutrophils

**DOI:** 10.3390/ijms22094726

**Published:** 2021-04-29

**Authors:** Ashley A. Stegelmeier, Maedeh Darzianiazizi, Kiersten Hanada, Shayan Sharif, Sarah K. Wootton, Byram W. Bridle, Khalil Karimi

**Affiliations:** Department of Pathobiology, Ontario Veterinary College, University of Guelph, Guelph, ON N1G2W1, Canada; astegelm@uoguelph.ca (A.A.S.); mahi.azizi@uoguelph.ca (M.D.); khanada@uoguelph.ca (K.H.); shayan@uoguelph.ca (S.S.); kwootton@uoguelph.ca (S.K.W.)

**Keywords:** type I interferon, neutrophils, antiviral responses, COVID-19

## Abstract

Interferons (IFNs) are induced by viruses and are the main regulators of the host antiviral response. They balance tissue tolerance and immune resistance against viral challenges. Like all cells in the human body, neutrophils possess the receptors for IFNs and contribute to antiviral host defense. To combat viruses, neutrophils utilize various mechanisms, such as viral sensing, neutrophil extracellular trap formation, and antigen presentation. These mechanisms have also been linked to tissue damage during viral infection and inflammation. In this review, we presented evidence that a complex cross-regulatory talk between IFNs and neutrophils initiates appropriate antiviral immune responses and regulates them to minimize tissue damage. We also explored recent exciting research elucidating the interactions between IFNs, neutrophils, and severe acute respiratory syndrome-coronavirus-2, as an example of neutrophil and IFN cross-regulatory talk. Dissecting the IFN-neutrophil paradigm is needed for well-balanced antiviral therapeutics and development of novel treatments against many major epidemic or pandemic viral infections, including the ongoing pandemic of the coronavirus disease that emerged in 2019.

## 1. Recognition of Viral PAMPs by Neutrophils

**Figure 1:**
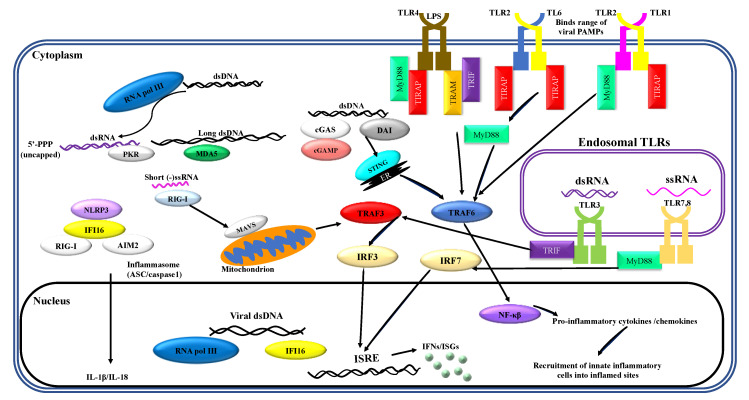
Virus-induced inflammatory responses. Recognition of viral pathogen-associated molecular patterns (PAMPs) by innate cells of the immune system results in inflammatory responses. Activation of innate leukocytes via pattern recognition receptors (PRRs) that recognize viral PAMPs in different cellular compartments gives rise to a number of intracellular signaling cascades, mediated by various interconnected adaptor proteins. This results in interferon regulatory factor (IRF)-mediated upregulation of interferons (IFNs) and interferon-stimulated genes (ISGs), as well as nuclear factor kappa-light-chain-enhancer of activated B cells (NF-κB)-mediated induction of inflammatory cytokines and chemokines. Furthermore, sensing of viral PAMPs by NOD-like receptor family pyrin domain containing 3 (NLRP3), retinoic acid-inducible gene I (RIG-I), absent in melanoma 2-like receptors (AIM2), and/or IFN-inducible protein 16 (IFI16) potentiates the formation of inflammasome complexes, which ultimately result in the induction of inflammatory cytokines such as interleukin (IL)-1β and IL-18. Other abbreviations: cGAMP: cyclic guanosine monophosphate–adenosine monophosphate, cGAS: cyclic guanosine monophosphate–adenosine monophosphate synthase, DAI: deoxyribonucleic acid (DNA)-dependent activator of interferon regulatory factors, ds: double-stranded, ER: endoplasmic reticulum, ISRE: interferon-sensitive response element, MAVS: mitochondrial antiviral signaling protein, MDA5: melanoma differentiation-associated protein 5, MyD88: myeloid differentiation primary response 88, NOD: nucleotide-binding oligomerization domain, PKR: protein kinase R, pol: polymerase, RNA: ribonucleic acid, ss: single-stranded, STING: stimulator of interferon genes, TIRAP: Toll/interleukin-1 receptor (TIR) domain-containing adapter protein, TLR: toll-like receptor, TRAF: tumor necrosis factor receptor–associated factor, TRAM: TIR-domain-containing adapter-inducing IFN-β (TRIF)-related adaptor molecule.

Neutrophils are the largest proportion of any cell subset within the innate immune system [1] and have traditionally been thoroughly characterized as an effective component of bacterial pathogen clearance. Research over the past decade has emphasized the expanding role of these innate cells in viral clearance [2,3,4]. Innate immune responses are initiated by recognition of PAMPs by a limited array of specific pattern recognition receptors (PRRs) expressed in and on sentinel cells. Recognition of viral PAMPs by PRRs—expressed by hematopoietic and non-hematopoietic cells of the immune system—results in the activation of intracellular signaling pathways, mediated by several interconnected adaptor proteins. Toll-like receptors (TLRs), which are an important class of PRRs, signal through a range of adaptor proteins. These virus-induced intracellular signaling pathways eventually converge on IFN regulatory factor (IRF)-mediated upregulation of IFNs and IFN-stimulated genes (ISGs) (Figure 1).

Neutrophils express a broad repertoire of PRRs and respond to PRR ligation during viral infection and inflammation. Neutrophils express all TLRs except for TLR3 [5]. Granulocyte-macrophage colony stimulating factor (GM-CSF), which controls different cell functions in inflammation, can promote neutrophil survival and trafficking; it can also upregulate oxidative burst, phagocytosis, and formation of extracellular traps [6], and increase both TLR2 and TLR9 expression in neutrophils [5]. TLR4, which recognizes lipopolysaccharide (LPS), was shown to be required for neutrophil migration to the lungs [7]. Neutrophils frequently travel to the lungs after a range of viral infections, including those caused by respiratory syncytial virus (RSV), highly pathogenic avian influenza virus, influenza A virus (IAV) [1], and vesicular stomatitis virus (VSV) [8]. However, neutrophils are still capable of killing a range of pathogens independent of TLRs [9]. For instance, it has been shown that neutrophil-derived IFN-γ is required for TLR-independent host protection against intracellular pathogens [10].

Studies were conducted on the interactions between viruses and neutrophil TLRs [11]. Neutrophils quickly upregulated TLR2 expression after exposure to IAV [12], and neutrophils treated with IAV increased their ability to phagocytize other pathogens. The single-stranded RNA recognition receptors, TLR7 and TLR8, were also involved in the neutrophil response to IAV [13]. TLR4 signaling in plasmid-transfected neutrophils resulted in expression of IFN-β [14]. Similar production of IFN- β was documented for a wide range of pathogens, including human adenovirus serotype 5. The TLR4 agonist LPS resulted in additional upregulation of IFN-β transcripts [14] and multiple research groups have elucidated a link between TLRs and the production of antiviral interferons. It is unclear if the TLR/type I IFN axis could somehow be modulated in a way to gain an appropriate antiviral immune response while minimizing tissue damage.

## 2. Interferons

Interferons are a large family of pleiotropic cytokines that play an essential role in host antiviral defenses. They have been classified into three distinct groups: type I, type II, and type III IFNs. These classifications are based on sequence identity, cognate receptors, biological functions, and sources of origin [15,16,17,18]. Almost all nucleated cells can produce type I IFNs. However, the natural IFN-producing cells, also known as plasmacytoid dendritic cells (pDCs), produce more type I IFNs on a per-cell basis than any other cells in the body [19]. Type I IFNs are produced upon viral infection and exert antiviral effects through interaction with the IFN-α/β receptor (IFNAR), which is expressed on almost all cells [20,21,22]. The type II IFN family has a single member, IFN-γ, that interacts with the interferon gamma receptor (IFNGR), which is expressed on a broad range of cell types. In contrast to type I IFNs, IFN-γ is mainly produced by leukocytes [23]. Type III IFNs include four subtypes: IFN-λ1 (IL29), IFN-λ2 (IL28A), IFN-λ3 (IL28B) and IFN-λ4. Like type I IFNs, they are induced by viruses. In contrast to the broad tropism of type I IFNs, the antiviral activity of type III IFNs appears to be more specialized for protection of epithelial cells [24]. The tissue-specific effects of these cytokines can be explained by the distribution of their receptor, IL28RA, which is mainly expressed on epithelial cell surfaces. These cytokines have been shown to be important, primarily in protection of the respiratory tract and the gut epithelium against viral infections [19,25,26,27,28,29]. Despite signaling through different receptors, type I and III IFNs trigger similar downstream signaling cascades and, therefore, mediate comparable biological functions [30,31].

**Figure 2:**
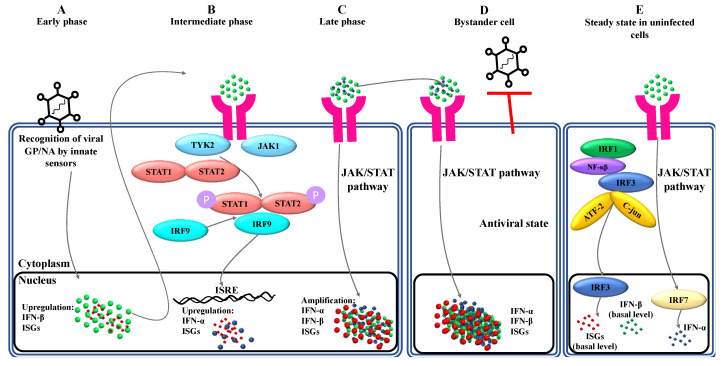
The Triphasic Model of Induction of Type I IFN Genes. (**A**) The expression of the interferon (IFN)-β gene is immediately upregulated following recognition of viral pathogen-associated molecular patterns (PAMPs). If the virus is successfully eliminated, no additional IFNs are induced. (**B**) Otherwise, further production of interferon regulatory factor (IRF)-3-induced IFN-β results in continual IFN-α/β receptor (IFNAR) signaling and thereby IRF-7-mediated upregulation of the IFN-α gene. (**C**) Subsequently, in the late phase of viral infection, the newly produced IRF7, in cooperation with IRF3, activates a positive feedback loop to amplify the induction of IFN-α/β genes to efficiently eliminate the invading virus. (**D**) Via paracrine interaction with IFNARs, type I IFNs create an antiviral state in bystander cells and, therefore, reduce or prevent virus spread to neighboring cells. (**E**) In a steady state, basal expression and signaling of IFN-β through IFNARs in uninfected cells is speculated to intrinsically provide protection against the potential for viral infections. Other abbreviations: ATF: activating transcription factor, GP: glycoprotein, ISGF: interferon-stimulated gene factor, JAK: Janus kinase, NA: neuraminidase, NF: nuclear factor, P: phosphate, STAT: signal transducer and activator of transcription.

A triphasic (early, intermediate, and late) model of type I IFN responses was proposed from extensive murine studies [32,33,34] (Figure 2). As depicted earlier, viral recognition by innate cells of the immune system resulted in the induction of intracellular signaling pathways, culminating in upregulation of IFN-β and ISGs [35,36,37,38] (Figure 2A)**.** Autocrine feedback of IFN-β with IFNAR on virus-infected cells mediated the intermediate phase of the response, beginning with the activation of the Janus kinase/signal transducer and activator of transcription (JAK/STAT) signaling pathway, leading to formation of an IFN-stimulated gene factor 3 (ISGF3) transcription factor complex, whose complex was composed of phosphorylated STAT1/STAT2 and IRF9. Binding of ISGF3 to IFN-stimulated response elements in the promoter region of ISGs upregulated numerous IFN target genes, including interferon regulatory factor 7 (IRF7). Like IRF3, activated cytoplasmic IRF7 translocated to the nucleus to induce the induction of IFN-α genes [39,40,41,42] (Figure 2B). The late phase of the IFN response was mediated by a positive feedback loop, through which IRF7 and IRF3 cooperated to induce robust induction of IFN-α and -β genes, respectively. This, in turn, led to the induction of numerous ISGs mediating type I IFN-driven antiviral responses [27,41,43,44,45,46,47] (Figure 2C). Amplification of IFN α/β genes in the late phase of the type I IFN response was associated with boosted pro-inflammatory responses, resulting in increased infiltration of type I IFN-producing-effector cells and thereby further amplification of inflammatory responses. Ultimately, type I IFNs not only promoted apoptosis of virus-infected cells but also prevented virus spread into uninfected neighboring cells (via paracrine interaction with IFNAR on the surface of those cells), decreased protein translation and synthesis, induced MHC class I and II expression, and degraded RNA [42,44] (Figure 2D). While IRF3 is essential for both early and late phases of the IFN response, IRF7 is a critical component of the positive feedback loop during the late stage of the IFN response. Unlike IRF3, which is constitutively expressed in uninfected cells, IRF7 is a transient, short-lived protein that is produced and phosphorylated in response to IFN-α/-β signaling within virus-infected cells [45].

In a steady state and in the absence of a viral invasion, the intrinsically expressed transcription factors IRF1, nuclear factor (NF)-κB, activating transcription factor 2 (ATF2)/c-Jun, and IRF3 induce a basal expression of IFN-β and a subset of their target genes known as ISGs [48,49,50,51]. Indeed, IRF3 was shown to be constitutively expressed in uninfected mouse embryonic cells. At this stage, IRF7 has been found to be expressed at a very low level as a result of constitutive, basal signaling of IRF3-induced IFN-β [41,50,52]. Given that IRF7 induces IFN-α gene expression, it has been speculated that a basal expression of IFN-α and -β may provide an uninfected cell with some level of intrinsic protection against an invading virus (Figure 2E). However, a constitutive and IRF-3/IRF-7-independent expression of IFN-α and -β genes in uninfected cells has been reported to enhance the positive feedback mechanism upon viral infection [42].

Type I IFNs have been implicated in the promotion of multiple viral [52,53] and nonviral infection-associated pathologies [54,55], as well as immune-mediated inflammatory diseases [56]. The beneficial viral clearance outcomes of type I IFN responses or detrimental consequences of sustained activation of immune responses have been found to be context-dependent; duration and magnitude of the responses appear to be critical factors [57,58,59,60,61,62]. In other words, a protective IFN response requires an intricate balance between stimulatory and modulatory responses. While it promotes effective clearance of infections, it also needs to facilitate a return to homeostasis.

Intrinsic low-level expression of ISGs during homeostatic, uninfected conditions, has been shown to protect hosts from viral infections [58]. Such constitutive expression of ISGs appears to determine susceptibility of a host to infection with viruses [63] such as IAV [64] and reovirus [65]. This can result in diminishing the accumulation of type I IFN response-inducing viral PAMPs, thereby reducing the risk of developing systemic inflammatory responses and associated immunopathology. This phenomenon was demonstrated in an in vivo study evaluating the treatment efficacy of IFN-α and IFN-λ in the setting of respiratory IAV infection [66]. Treatment with exogenous IFN-α exacerbated infection-associated pathology. Specifically, viral replication was limited, but concentrations of inflammatory cytokines in bronchoalveolar lavage fluids were augmented and associated with increased infiltration of inflammatory cells, including pDCs and inflammatory monocytes, into lungs, and increased apoptosis of airway epithelial cells. On the other hand, administration of exogenous IFN-λ was shown to reduce viral spread without inflammatory side effects. This protective effect was attributed to restriction of IAV replication and IFN-λ-induced responses of pulmonary epithelial cells, as well as an inability of IFN-λ, unlike IFN-α, to directly stimulate cells of the immune system.

Regarding host IFN-dependent and -independent antiviral responses, peroxisomal and mitochondrial antiviral-signaling proteins have been shown to function in a consecutive manner towards establishment of type I IFN-dependent and-independent antiviral states, respectively. In the event of viral invasion, peroxisomal antiviral-signaling proteins launch an immediate antiviral cascade from the membranes of peroxisomes leading to induction of early, IFN-independent genes which restrain viral replication until a robust and sustained antiviral response is initiated via MAVS, with delayed kinetics of IFN-α/-β gene expression. In addition to this, the IFN-independent peroxisomal antiviral-signaling pathway is thought to be important in restraining viruses that interfere with cellular type I IFN responses such as VSV. In line with this notion, cells expressing only MAVS showed the same susceptibility to VSV infection as MAVS-deficient cells [67,68]. Additionally, epithelial cells have an antiviral pathway that is activated prior to IFN pathways. Neutrophils can respond to viral infections using a CXCR-3-dependent mechanism after epithelial CXCL10 is released [69].

A consecutive order of local and systemic antiviral responses has also been reported in the case of IAV that preferentially invades and replicates in the epithelial cells of mammalian upper respiratory tracts [70]. IFN-λ has been shown to establish a local, noninflammatory defense against IAV within the epithelial cells, which could be followed by a systemic, inflammatory, and potentially pathogenic type I IFN response [71]. The IFN-mediated inflammatory responses were then proposed to occur predominantly in compartments beyond the epithelial cells. Consistent with this proposition, IFN-λ was recently found to be more important than IFN-α/β in preventing the spread of influenza viruses from the upper respiratory tract into the lungs of infected mice [72]. In addition, ISGs were reported to exert antiviral effects, not only via both IFN-mediated and IFN-independent pathways [73], but also independently of these cytokines [48,74,75,76,77,78,79]. These findings together suggest how early, local antiviral mechanisms controlling early stages of a viral infection can prevent pathological outcomes of systemic type I IFN responses against viruses.

## 3. Regulation of IFN Signaling in Neutrophils

Neutrophils’ ability to produce IFNs in conjunction with recognition of viral PAMPs suggests that they are critical for innate antiviral host defenses. Using a range of stimulatory compounds, researchers demonstrated that messenger RNAs encoding IFN-α, -β, and -γ were constitutively expressed in neutrophils [5]. The presence of type I IFNs reduces the concentration of lipid A, a TLR4 agonist that is required to induce TRIF-dependent genes, demonstrating a link between TLR4 and IFNs [80]. Neutrophils can use helicase recognition to activate a robust antiviral response [81]. The viral double-stranded RNA mimetic poly(I:C) can be recognized by neutrophils, despite them not possessing TLR3. Constitutive expression of MDA5 and RIG-I aids neutrophils in recognizing the viral genetic material and subsequently producing type I IFNs, IFN-responsive genes (IRGs), and immunoregulatory cytokines. These findings were reinforced in experiments using encephalomyocarditis virus in MDA5-deficient mice, which have a reduction in IFN-β production [81].

**Figure 3:**
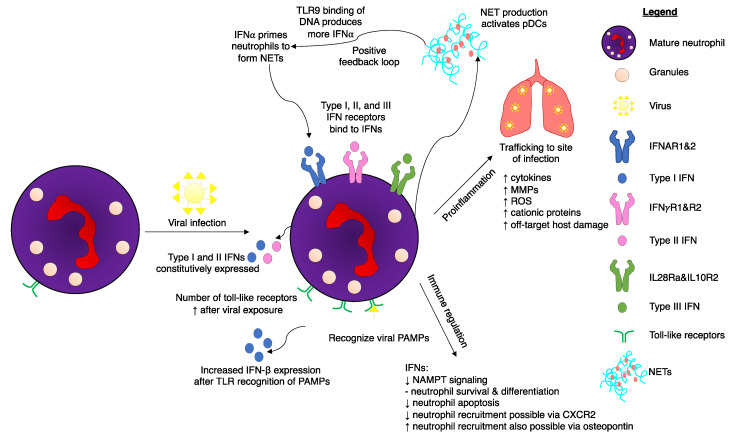
Interferon (IFN)-mediated regulation of neutrophils upon viral infection of a host. Viral infections induce many changes to neutrophil biology. Toll-like receptor (TLR) expression increases after viral exposure, resulting in increased production of type I interferons (IFNs). Neutrophils possess receptors for all three interferon subsets. A positive feedback loop occurs during the production of neutrophil extracellular traps (NETs). IFNα results in NET production, which in turn activates plasmacytoid dendritic cells (pDCs). Binding of deoxyribonucleic acid (DNA) from the NETs to TLR9 produces more IFNα, which, in turn, can result in excessive NET production. IFN pathways result in both a proinflammatory response and immunoregulation. Although neutrophils are integral for hosts to successfully eliminate viral infections, certain viruses have adapted mechanisms to hijack the IFN response to cause unwanted neutrophil-induced host damage. Excessive production of cytokines can lead to fatal immune-mediated overreactions to the viral threat. Other abbreviations: CXCR2: CXC chemokine receptor 2, MMP: matrix metalloproteases, NAMPT: nicotinamide phosphoribosyltransferase, ROS: reactive oxygen species.

Mature neutrophils are predominantly responsible for neutrophil-mediated IFN responses, as immature neutrophils do not express IFNARs and have lower IRG expression levels [82]. Immature neutrophils are also incapable of effectively phosphorylating STAT1 and are not primed effectively by IFNs. Likewise, studies of immature neutrophil gene regulation illustrated limited IFN ability to control immature neutrophil proliferation. Although IFNs did not have an effect on immature neutrophils, IFN-α does influence their precursor hematopoietic stem cells by activating dormant cells [83]. In contrast, mature neutrophils express genes to enable them to respond to both type I and II IFNs [82]. IFN-α primes mature neutrophils, enabling them to form neutrophil extracellular traps (NETs) to bind to pathogens (Figure 3). In a positive feedback loop, these traps—which are composed primarily of DNA, high mobility group box protein 1 (HMGB1), and the cathelicidin antimicrobial peptide LL37—subsequently activate pDCs, which in turn produce more IFN-α via DNA binding to TLR9 [84]. Interferon-deficient mice have reduced production of NETs and reactive oxygen species (ROS), while recombinant IFN-β treatment restored NETosis [85]. Controlling this feedback loop may be a method warranting further examination in diseases that are exacerbated by excessive formation of NETs.

Certain viruses are capable of infecting neutrophils. During IAV infection, neutrophils initiate a multifaceted immune response. Type I IFNs are expressed, along with ISGs and upregulation of PRRs [13]. Viral entry is required for this to occur, but replication is not essential. The virulent H3N2 influenza strain also infects neutrophils and induces a robust type I IFN signaling and regulatory response starting at three hours post-infection [11]. Lungs experiencing viral infection have a different immunological environment compared to bacterial lung infections, composed of type I IFNs and their resulting ISGs. It thus follows that neutrophils entering this virus-conditioned microenvironment would respond differently than they would to a bacterial infection. Viruses also possess genes to suppress type I IFNs to mediate their survival. IAVs express a nonstructural protein (NS1) that prevents induction of IFN-β [86]. Experiments in ferrets using NS1 from the pandemic-causing strain of IAV from 1918 determined that this protein significantly delayed the type I IFN response [87]. Moreover, the USSR/90/77 strain of IAV mediated a less pronounced delay in the IFN response. Additional research in ferrets showed mild influenza infections had robust innate responses, while severe disease was associated with reduced type I and type II IFN responses [88]. A genetically altered variant of IAV with NS1 deleted restored the IFN-α and IFN-β responses, coupled with increased NF-κB activation [89]. Influenza virus-infected neutrophils initiated the adaptive immune system by transitioning into antigen-presenting cells and subsequently activating effector antiviral CD8^+^ T cells [90]. Neutrophil depletion decreased the magnitude of virus-specific CD8^+^ T cells, although it did not impact T cell trafficking in the context of a pulmonary influenza infection [91].

Type I IFNs are integrally intertwined in most aspects of neutrophils’ existence, mediating both neutrophil production and cellular regulation. Type I interferons regulate nicotinamide phosphoribosyltransferase (NAMPT) signaling, which, in turn, is involved in survival and maturation of neutrophils [92]. Specifically, IFNs suppress NAMPT, as demonstrated in IFN-deficient animal models. Deficiency of IFN leads to an increase in NAMPT during neutrophil progenitor maturation in the bone marrow. During development, NAMPT increases early progenitor survival and later slows down neutrophil differentiation. During later life stages, when mature neutrophils are recruited to infected regions, IFN—alongside G-CSF and TNF—prolongs neutrophil survival [4]. Interferon-α delays neutrophil apoptosis by inducing cellular inhibitors of apoptosis 2 (cIAP2) via STAT in a similar manner to G-CSF [93]. Synthesis of cIAP2 is dependent on Janus kinase 2-STAT3 activation. Type I IFNs downregulate G-CSF, which is involved throughout the neutrophil lifecycle [94,95,96]. G-CSF causes STAT3-dependent changes within the bone marrow, influencing neutrophil migration [97]. By downregulating key neutrophil migratory control signals, IFN production can control the magnitude of neutrophil-mediated responses to viral infections. Interferon-β initiates phosphatidylinositol-3 kinase-dependent survival for neutrophils, thus preventing apoptosis [98]. In the context of cancers, IFN-β is needed to maximize neutrophil cytotoxicity [95].

Neutrophils can produce type II IFNs under multiple conditions. During renal ischemia-reperfusion injuries, neutrophils produce IFN-γ, a phenomenon that is dependent upon activation of natural killer-T cells in the kidneys within three hours of reperfusion [99]. IFN-γ is also produced by Gr-1^+^CD11b^+^ cells in the context of early islet graft rejection of the pancreas, which are again reliant on NKT cells [100]. Pathogens are also capable of initiating IFN-γ responses by neutrophils, as observed in *Streptococcus pneumoniae* experiments [101]. Clearance of pathogens, mediated by neutrophil-derived IFN-γ, is reliant on nicotinamide adenine dinucleotide phosphate (NADPH) oxidase, Ras-related C3 botulinum toxin substrate 2 (Rac2), and Hck/Lyn/Fgr Src family tyrosine kinases. Type II IFN production within neutrophils requires these compounds to be produced. NETs are a proposed clearance mechanism [102]. Detailed analyses have illustrated that MyD88 is also critical for IFN-γ production by neutrophils, although TLRs and TRIF are not apparently involved [101]. Neutrophils subsequently can respond to IFN-γ by upregulating expression of genes and oxidative burst capabilities. Clearly, the traditional definition of neutrophils as terminal phagocytes has been altered by research demonstrating fine-tuned neutrophil protein synthesis in response to external stimuli [103].

## 4. Walking on a Knife Edge: Host Neutrophils Associated with both Protection and Severe Adverse Events following Viral Infection

Early recruitment of innate inflammatory cells into virus-infected sites is required, not only for promoting inflammatory responses, but also for tissue regeneration and establishment of a homeostatic state after ultimate control of an infection. Neutrophils are the first subset of leukocytes mobilized to sites of virus infection [104]. In IAV-infected mice, pulmonary accumulation of neutrophils was observed one day post-infection and persisted for seven days [105]. Neutrophils trafficking to inflamed tissues are followed by infiltration of other cells of the immune system, including macrophages, dendritic cells, natural killer cells, and B and T lymphocytes [106,107,108]. Neutrophils and macrophages are major effector cells involved in promotion of inflammatory responses against a viral infection. They are also involved in immunomodulation and establishment of a homeostatic state following successful clearance of viruses [90,109,110,111,112,113]. In addition to infiltrating cells, residential cells—such as lung- and liver-resident myeloid cells, particularly alveolar macrophages and Kupffer cells—play a major role in promotion of antiviral responses and restoration of lung homeostasis following clearance of a viral infection. They do so by restraining lung-infiltrating inflammatory cells and subsequently aiding in resolution of inflammation [111,112,114,115,116].

Despite their critical role in promotion of host antiviral responses, excessive infiltration of inflammatory cells into virus-infected and/or inflamed sites and persistent production of inflammatory cytokines create an extreme inflammatory environment that can lead to a severe condition where an exaggerated host immune response (rather than viral cytopathic effects) can cause fatal tissue/organ damage [117,118,119,120,121,122]. Comparisons of HIN1 and H3N2 infections versus highly pathogenic pandemic IAV strains, including H5N1, revealed that early excessive inflammatory responses and massive infiltration of proinflammatory cells into the lungs were determinant factors in lethal outcomes of infection with the H5N1 strain, compared with nonlethal H5N1 and seasonal IAV strains [123,124,125]. As mentioned, myeloid cells, including neutrophils and monocytes, are amongst the first leukocytes that are recruited to sites of infection. They are key contributors to overly robust host inflammatory responses that can cause substantial tissue/organ damage after some viral infections [126,127,128,129,130]. Moreover, a massive increase in concentrations of inflammatory cytokines in plasma (particularly IL-6) and chemokines that attract neutrophils (e.g., CXCL 8) and monocytes (e.g., CXCL10 and monocyte chemoattractant protein 1 [MCP-1]) were found to underlie the fatal outcome of an array of viral infections [65,128,131,132].

A range of inflammatory mediators, including cytotoxic cytokines, ROS, lipid mediators, and cationic proteins, released by neutrophils and macrophages were reported to contribute to tissue damage during viral infections (Figure 3) [133]. Recent data demonstrated that matrix metalloproteases (MMPs) can cause irreparable pulmonary damage during IAV infections [129,131]. MMPs are proteolytic enzymes primarily produced by neutrophils [132], and are involved in remodeling of the extracellular matrix during physiological and pathological events. Under inflammatory conditions, however, substantial release of MMPs by infiltrating leukocytes can contribute to pathogenesis, including in the pulmonary system [134,135]. Accordingly, substantial release of MMP9 and membrane type I (MT1)-MMP/MMP-14 enzymes by neutrophils and myeloid cells, respectively, were reported to significantly contribute to IAV-induced pathology and mortality [133,136]. The importance of neutrophil-derived MMP9 was already addressed in lung pathogenesis secondary to induced pancreatitis in rats [137]. Fatal consequences of infection with IAV were shown to be independent of viral or bacterial burden, arising instead from host failure to tolerate or repair the massively damaged lung tissue.

Host innate antiviral responses are largely controlled by type I IFNs, which exert their antiviral and immunomodulatory effects by interaction with IFNAR [137,138,139,140]. As intracellular obligatory parasites, viruses have strategies to compromise host type I IFN-mediated antiviral responses. Despite their well-established protective roles against invading pathogens [141,142], virus-induced aberrant type I IFN responses have been associated with toxic inflammatory responses and development of immunopathology [58,63,77,143,144,145,146,147,148,149,150,151]. IAV-induced type I IFN responses have been associated with cytokine storms, characterized by high levels of inflammatory cytokines/chemokines and massive infiltration of inflammatory cells resulting in widespread tissue damage and increased fatalities [150]. Indeed, cytokine storms occur when viruses interfere with transcriptional responses of a range of both chemokines and cytokines [152]. While the pathogenic role of type I IFNs has been demonstrated in the setting of IAV infection [64,153,154], protective virus-induced type I IFN responses have also been reported in the context of infection with the IAV strain A/Puerto Rico/8/34 (PR8/H1N1). PR8/H1N1-infected IFNAR-knockout mice experienced severe lung inflammation and pathology characterized by massive infiltration of neutrophils mediated by keratinocyte chemoattractant-producing Ly6C^hi^ monocytes [155]. Viruses have a myriad of strategies to sabotage host antiviral defenses. In turn, hosts mount antiviral responses through a wide variety of parallel pathways. The strategy used by an infecting virus to interfere with host immunity seems to influence the antiviral pathway or pathways utilized by a host [59,78,156].

In a C57BL/6 murine model of influenza infection, expression of high concentrations of IFN-α and IFN-β in bronchoalveolar lavages was associated with high morbidity and pulmonary damage. Depletion of pDCs and inflammatory monocytes decreased disease severity, while depleting neutrophils did not significantly alter disease progression [58]. In contrast, in a murine model of the moderately virulent HKx31 influenza virus, neutrophils were quickly recruited to both the upper and lower respiratory tract [157] and reduced disease severity. Neutrophils can inhibit influenza replication [153] in vivo in both tumor-free and tumor-bearing mice. Indeed, neutrophil depletion studies showed that their absence led to exacerbated inflammation, edema, weight loss and ultimately death [154] after infection with IAV H3N2. This was in part due to the ability of neutrophils to limit influenza infection by limiting early stage IAV replication and reducing vascular permeability. However, neutrophils are not always associated with a positive prognosis after infection with influenza viruses. Transcriptome analysis revealed that the most severe clinical cases of influenza had high neutrophil burdens [158]. In the acute stages of infection (and in cases of mild disease), expression levels of interferon-inducible genes and type I IFNs were elevated. Similar findings have been made with other pathogens. For example, neutrophils had complex interactions with IFNs in the case of the bacterium *Francisella tularensis* [156] and *Leishmania amazonensis* [159]. Studies with IFNAR-knockout mice showed that neutrophils were protective up to a certain threshold [85]. If rampant accumulation occurred, neutrophil-associated damage commenced. Complete depletion of neutrophils can also be detrimental. As such, moderate neutrophilia is likely ideal for the majority of pulmonary infections.

A notable study explored the interactions of type I interferons and neutrophils during viral pneumonia [64]. Using IFNAR-knockout mice, it was demonstrated that defective IFN signaling led to an increase in neutrophil infiltration due to Ly6C^int^ monocytes preferentially producing the neutrophil chemokine KC, in contrast to wild type murine Ly6C^hi^ monocytes producing MCP-1. Knockout mice, therefore, had excessive trafficking of neutrophils into the lungs, causing them to surpass a critical threshold and cause tissue damage [64]. An intact type I IFN system is thus integral for fine-tuning neutrophil antiviral responses, as IFN was required to correctly generate Ly6C^hi^ monocytes.

Another virus that has been extensively studied to elucidate neutrophil biology is RSV. RSV causes severe lower respiratory tract infections in infants. Neutrophils are abundant in pulmonary airways during RSV infections. A thorough transcriptome analysis of pulmonary versus blood-derived neutrophils suggested IL-6 and ISGs are upregulated during RSV infections [160]. The authors of the study did not specify if the IFN response was classified as type I or II. This distinction should have been made because upregulated ISGs are common to both pathways. A multitude of transcriptome studies in models of RSV infections have demonstrated that over 207 transcripts related to IFN signaling were upregulated after exposure to the virus. High IFN transcript expression was independent of the nasopharyngeal microbiota present [161] and could even predict disease severity [162]. Likewise, the study was unable to comment on whether type I or type II IFNs were the dominant response. Dysregulated IFN responses were higher in infants with RSV compared to children aged 5-17 with the same disease and were driven by type I IFN-associated pathways such as TNF, IL-6, and triggering receptor expressed on myeloid cells-1 (TREM-1) [163]. In contrast to some influenza studies, severe cases of RSV infections had higher levels of neutrophil markers in the mucosal lining fluid of the nose obtained from 55 infants admitted to hospital [164]. The neutrophil-associated genes defensin-a1, cathelicidin, granzyme, and antimicrobial peptides clustered with type I IFNs in a gene expression study, although their expression levels did not stratify along patient disease severity [164]. The severest cases had lower type I IFN-associated gene expression, potentially indicating a protective effect endowed by a biologically optimal level of interferon response.

There is evidence that IFN-β can control the magnitude of neutrophil musculoskeletal infiltration. Indeed, IFN-β-knockout mice infected with Chikungunya virus (CHIKV) had four times more neutrophils infiltrate musculoskeletal tissue compared to controls that expressed IFN-β [165]. While IFN-β controlled neutrophil-mediated downstream inflammation, IFN-α prevented early CHIKV replication and subsequent dissemination, as quantified via plaque assay to measure viral titer. IFN-β knockout mice did not have higher concentrations of the neutrophil-attracting chemokines CXCL1 or CXCL2. Instead, they had decreases in a multitude of cytokines, including TNF, CXCL -9, CXCL-10, CCL-2, CCL-3, and CCL-5 [165]. Therefore, in mice with intact IFN-β, higher neutrophil infiltration was able to occur, despite no measured elevation in neutrophil-attracting chemokines. The authors suggested that IFN-β may have had an effect on nonhematopoietic cells, resulting in eventual increases of neutrophil recruitment to the inflamed sites, although no exact mechanism was determined. Studies of zebrafish infected with CHIKV were used to elucidate that type I IFNs were predominantly produced by neutrophils to control infection [166]. Neutrophil depletion exacerbated disease progression and increased the viral load (as measured by CHIKV transcripts). It was also demonstrated that CHIKV induced NETs via both ROS production and TLR7 activation. The effect was not universal for all viruses tested, as Dengue virus (DENV2) and Zika virus (ZIKV) were incapable of inducing NETs [167]. Reducing the DNA-based NET load by using DNases restored CHIKV titers in IFNAR-knockout mice.

Aside from pulmonary infections, excessive neutrophilia can cause damage when these cells infiltrate regions of the brain. Thus, the immune system also has regulatory components that are driven by type I IFNs to mitigate damage from the innate effector mechanisms. Interferons inhibited neutrophil recruitment by downregulating the chemokine CXCR2 [168] in a herpes simplex virus type 1 model. CXCR2 was downregulated in the sensitive ganglia region, while upregulation on the skin directed neutrophils to a location less sensitive to off-target inflammatory pathology. IFN-β controls recruitment of neutrophils by regulating CXCR2 ligands. In contrast to herpes virus infections, neutrophils were shown to be beneficial to the host by reducing viral load during ZIKV infections, which was associated with diminished ZIKV-induced neurological damage [169]. Interferon α/β receptor-knockout mice (strain AG129) had exacerbated hindlimb motor impacts according to the Basso scale, which assesses tail position, joint movement, and limping. Decreases in hindlimb mobility were predominantly due to spinal cord myelitis rather than peripheral neuropathy or upper motor neuron disease and were inversely proportional to neutrophil infiltration.

Fish models have been useful for studying the impact IFNs have on neutrophils. Particularly, studies of gilthead seabream (*Sparus aurata* L.) and zebrafish (*Danio rerio*) have yielded valuable results. Sindbis virus (SINV) is a virion that induces type I IFN responses, and sometimes results in bacterial coinfection [170]. Experiments using sequential SINV and *Shigella flexneri* bacterial infection in zebrafish larvae demonstrated that neutrophils were essential to prevent bacterial coinfections. Whole-body analysis showed an increase in neutrophils when SINV was the only pathogen, but a SINV-*Shigella* coinfection dramatically reduced neutrophil numbers, in contrast to an infection of only *Shigella* [170]. Primary infection caused by the virus interfered with the neutrophil ability to phagocytize the subsequent bacterial infection. SINV induced a strong type I IFN response, and, in the future, the authors plan on investigating the role IFN has on neutrophil populations during viral and bacterial coinfections. Furthermore, gilthead seabreams have proven useful in the study of ISG15, which is induced by type I IFNs [171]. Infections with viral hemorrhagic septicemia virus or striped jack nervous necrosis virus increased ISG15 transcript numbers after 12 h, peaking at 24 h [171]. ISG15 was upregulated by both viral infections and type I IFN stimulation [172]. This immune modulatory protein activates monocytes and induces IFN- γ production from T cells [173]. Although seabreams like those used in the above model [171] do not possess neutrophils, they have acidophylic granulocytes, which function similarly and can produce ISG15. Thus, the type I IFN system is conserved in influencing neutrophil-like behavior during viral infections in nonmammalian model organisms.

Neutrophils can also be infected by West Nile virus (WNV), which controls the cells, akin to a Trojan horse, and causes them to enter the brain and increase viral burdens [174]. Intracellular osteopontin (iOPN) is a protein that is produced by most leukocytes, including neutrophils. It helps to amplify type I IFN responses upon TLR7 and TLR8 activation [175]. Intriguingly, iOPN facilitates WNV neuro-infiltration, as OPN-knockout mice had lower burdens of WNV-infected neutrophils infiltrating the brain [174]. This may constitute one mechanism whereby a virus hijacks the type I IFN system to aid viral spread. In contrast, iOPN is critical in the prevention of severe VSV infections in mice [175], which it accomplishes by stabilizing TRAF3.

One outcome of the interactions between type I IFNs and neutrophils during viral infection is ROS production. Hydrogen peroxide can be generated via NADPH oxidase isoform 2 (NOX2), and it negatively regulates type I IFNs [176]. If NOX2 is inhibited, concentrations of type I IFN are increased and, consequently, IAV-induced lung pathology diminishes [177]. It was shown that Cgp91ds-TAT, a NOX2 inhibitor, reduced pulmonary neutrophil counts by ~50% in a mouse model of IAV [177]. Therefore, excessive amounts of endosomal-generated ROS can be a damaging factor, which may steer neutrophils from a protective to a damaging role during pulmonary infections [178]. Nevertheless, in optimal quantities, it can destroy pulmonary pathogens and induce NET formation [179].

## 5. The importance of Neutrophil Activation/Dysregulation of Type I IFN Responses in COVID-19 Patients

A global pandemic was declared by the World Health Organization in March 2020 to address the severe acute respiratory syndrome coronavirus 2 (SARS-CoV-2). The first cases of viral infection were reported in Wuhan, China in 2019 [180]. In some patients, this virus can cause an array of signs and symptoms, including severe pulmonary damage. The disease is commonly referred to as Coronavirus disease 2019 (COVID-19). The lungs of patients with severe disease exhibit a high neutrophil burden [181]. A cell subset defined as CD16^int^CD44^low^CD11b^int^ low-density inflammatory band closely matched patient disease status. These neutrophils displayed robust cytokine production and phagocytosis which, in turn, provoked pulmonary damage [181]. Neutrophils are thus an important cellular subset to monitor in patients with COVID-19. Studies in macaques revealed that infected lungs had significant increases in neutrophil degranulation and release of Type I IFNs [182], coupled with a higher neutrophil:lymphocyte ratio. Indeed, cells infected with SARS-CoV-2 expressed chemokines [183] such as CXCL1/2/3/5/8, which would, in turn, attract neutrophils and initiate a downstream cytokine storm. Based on these results, one could speculate that depleting neutrophils below a certain threshold may constitute a therapeutic option in patients with COVID-19.

There is mounting evidence that NETs may increase the severity of COVID-19 [181,184]. Formation of NETs could lead to excessive blood clotting because they increase coagulation and activate platelets [185]. Type I IFNs enhance NETosis [186] via the feedback mechanism between IFN-α and NETs described earlier in this review [84]. It is possible, therefore, that unchecked Type I IFNs could exacerbate neutrophil infiltration and NETosis.

Middle East respiratory syndrome (MERS) is caused by a β-coronavirus that is within the same genus as SARS-CoV-2 [187,188]. A study into MERS-CoV infections determined that the type I IFN response was closely related to the survival outcomes in a BALB/c murine model [189]. IFNs provided a protective response in mice. Blocking type I IFNs led to an increase in lung neutrophils, poor T cell responses measured by N99 and S1165 epitopes, and a reduction in viral clearance. If IFN-β was administered too late, it was incapable of reversing damage. These findings highlight the importance of admitting patients to hospitals in a timely fashion. Administering therapies only to the most severely ill patients may mean that those treatments will be too late to effectively mitigate damage, compared to treating earlier stages of coronavirus infection.

## 6. Neutrophils Respond to Type I and Type III IFNs to Regulate Viral Infections

Induced during viral infections, type I and type III IFNs share many properties, including activation of shared signaling pathways and transcriptional programs [190]. Type III IFNs steer the immune system towards a T helper 1 (Th1)-biased intracellular response [191] and interact with interleukin 10 receptors beta subunit (IL10R2) and interleukin 28 receptors alpha subunit (IFNLR1) when mounting antiviral responses. Type III IFNs are encoded by four genes in humans; IFN-λ1, IFN-λ2, IFN-λ3 and IFN-λ4. Their cognate receptors are predominantly located on epithelial cells [32], with high concentrations thus generated in the gastrointestinal tract and lungs. There is a close association between type III IFNs and neutrophils in mounting an antifungal response [192]. In a study, murine models with type I, III, or both receptors knocked out were all susceptible to fungal Aspergillus fumigatus infections. Mice with both IFNAR and IFNLR1 double knockouts (missing both type I and III responses) had pronounced decreases in lung neutrophil counts in a cumulative manner and, consequently, reduced NET production. The type I and III pathways are not completely redundant; double knockout mice performed worse than either single knockout scenario. Type I knockouts performed better than Type III, indicating type III IFNs contributed more to the antifungal response. Administering IFN-α, IFN-γ, or adoptive transfer of CCR2^+^ monocytes improved the neutrophil transcript profile. Neutrophils upregulated 887 genes after pulmonary *Aspergillus* infection, but this transcriptome response was dampened in neutrophils obtained from mice with CCR2 depletions. This study used numerous techniques, including knockouts, depletions, and transcriptomics, to demonstrate that neutrophils were an essential cell subset to mitigate fungal growth. Neutrophils express a high level of IFNLR1 [193], and as a consequence are tightly linked to type III IFN-driven immune responses. Administering IFN-λ was also effective in preventing excessive neutrophil infiltration and inflammation for collagen-induced arthritis [193]. In contrast to different mouse models of infectious diseases where neutrophils responded to IFN-λ to modulate responses, ex vivo experiments utilizing human neutrophils demonstrated that there is a low level of IFN-λR1 expression [194]. Therefore, human neutrophils appear to be less responsive to IFN-λ3 than their murine counterparts.

Type I & III IFNs are both able to mount effective antiviral responses. A murine neonatal study using simian rotavirus revealed that this virus was controlled by type I and III IFNs working in conjunction to limit replication in the gastrointestinal tract [33]. In contrast, a murine strain of rotavirus relied on the adaptive immune response without the need for either type of IFN. Intriguingly, mature mice had reduced capacity for type I IFN responses compared to neonates, although the type III IFN response remained intact, suggesting that the relative importance of each type of IFN changes as a host matures [33]. Similar models have demonstrated that, although type I and III IFNs have identical downstream signal transduction pathways, gastrointestinal viral infections in neonates can be prevented using IFN-λ therapies but not type I IFNs [30]. The importance of the innate immune system in combatting gastrointestinal viral infections was recapitulated in a murine study of norovirus [195]. IFN-λ was induced by the viral capsid protein and effectively controlled enteric infection, compared to type I IFNs being most effective during systemic infection. Supporting this observation, STAT1 was more important than IFNAR for controlling replication within the colon [195]. Although the studies mentioned above did not analyze neutrophils, it is possible that neutrophils were mechanistically contributing to IFN-λ-mediated efficacy, given their ability to mitigate fungal infections in concert with IFN-λ. Neutrophils are a critical cell subset to analyze when comparing the influence of type I and III IFNs, because they possess the receptors to react to both cytokines [77]. Future studies would benefit from specifically analyzing neutrophils to determine if their contribution extends to viral gastrointestinal infections.

## 7. Concluding Remarks

Host innate antiviral responses are largely controlled by type I IFNs signaling through the IFNAR. Viruses, as intracellular obligatory parasites, have a myriad of strategies to compromise host type I IFN-mediated antiviral responses [78]. This review investigated the roles of IFNs and neutrophils in cytokine responses to viral infections. The knowledge that the majority of cells in the body express the type I IFN receptor while epithelial cells and neutrophils preferentially express the type III IFN receptor indicates that neutrophils must be a critical cell subset that contributes to cooperation/regulatory talk between both IFN types during antiviral responses. These innate cells are closely intertwined with IFN signaling and production, and the magnitude of the neutrophil response often dictates whether these cells will aid or damage the host in the context of a viral infection. These interactions may enable future researchers to fine-tune the balance between neutrophil-mediated antiviral effects and undesirable host damage, to improve the next generation of antiviral therapies. Evidence suggested a crucial role for neutrophils in the pathogenesis of COVID-19, although less is known about the regulatory function and immune modulation of these cells in that context. Regardless, consideration could be given to modulating neutrophilia to treat patients with COVID-19. Future research that investigates the role of IFNs in antiviral responses would be strengthened by delving into a detailed analysis of neutrophil biology. In particular, there is scant data in scientific literature on the interactions between IFNs across various neutrophil subsets, and what variations occur because of host age and sex. A thorough understanding of how these parameters shape neutrophil-driven proinflammatory and regulatory responses may contribute to advancing the next generation of antiviral therapies. 

## Data Availability

Not applicable.

## Abbreviations

ATF2	activating transcription factor 2
cGAMP	cyclic guanosine monophosphate–adenosine monophosphate
cGAS	cyclic guanosine monophosphate–adenosine monophosphate (GMP–AMP) synthase
CHIKV	Chikungunya virus
COVID-19	Coronavirus disease 2019
DENV2	Dengue virus
DNA	deoxyribonucleic acid
GM-CSF	granulocyte-macrophage colony stimulating factor
HMGB1	high mobility group box protein 1
IAV	influenza A virus
IFN	interferon
IFNAR	IFN-α/β receptor
IFNGR	interferon gamma receptor
IFNLR1	interleukin 28 receptor, alpha subunit
IL	interleukin
IL10R2	interleukin 10 receptor, beta subunit
iOPN	intracellular osteopontin
IRAK	interleukin-1 receptor-associated kinase
IRF	IFN regulatory factor
ISG	IFN-stimulated genes
ISGF3	IFN-stimulated gene factor 3
IRG	interferon responsive genes
JAK	Janus kinase
LPS	lipopolysaccharide
MAVS	mitochondrial antiviral signalingm
MCP-1	monocyte chemoattractant protein 1
MDA-5	melanoma differentiation-associated protein
MMP	matrix metalloproteases
MyD88	myeloid differentiation primary response protein 88
NADPH	Nicotinamide adenine dinucleotide phosphate
NET	neutrophil extracellular traps
NF	nuclear factor
NOD	nucleotide-binding oligomerization domain
NOX2	NADPH oxidase isoform 2
PAMPs	pathogen-associated molecular patterns
pDCs	plasmacytoid dendritic cells
PRR	pattern recognition receptors
RIG-I	retinoic acid-inducible gene
RNA	ribonucleic acid
ROS	reactive oxygen species
RSV	respiratory syncytial virus
SARS-CoV-2	severe acute respiratory syndrome coronavirus 2
SINV	Sindbis virus
STAT	signal transducer and activator of transcription
STING	stimulator of interferon genes
Th1	T helper 1
TIR	Toll/interleukin-1 receptor
TIRAP	Toll interleukin-1 receptor-associated protein
TLR	Toll-like receptor
TNF	tumor necrosis factor
TRAF	TNF receptor associated factor 3
TRAM	TRIF-related adaptor molecule
TREM-1	Triggering receptor expressed on myeloid cells-1
TRIF	TIR-domain-containing adapter-inducing interferon-β
VSV	vesicular stomatitis virus
ZIKV	Zika virus
